# FTX contributes to cell proliferation and migration in lung adenocarcinoma via targeting miR-335-5p/NUCB2 axis

**DOI:** 10.1186/s12935-020-1130-5

**Published:** 2020-03-23

**Authors:** Xiaodong Huo, Huixing Wang, Bin Huo, Lei Wang, Kuo Yang, Jinhuan Wang, Lili Wang, Haitao Wang

**Affiliations:** 1 Department of Oncology, The Second Hospital of Tianjin Medical University, No. 23 Pingjiang Road, Hexi District, Tianjin, 300211 China; 2 Pain Management Center, The Second Hospital of Tianjin Medical University, Tianjin, 300211 China; 3 Central Laboratory/Tianjin Research Institute of Urology, The Second Hospital of Tianjin Medical University, Tianjin, 300211 China

**Keywords:** FTX, miR-335-5p, NUCB2, Lung adenocarcinoma

## Abstract

**Background:**

Extensive studies revealed that long non-coding RNAs (lncRNAs) could act as a regulator in tumors, including lung adenocarcinoma (LUAD). LncRNA FTX transcript, XIST regulator (FTX) has been reported to regulate the biological behaviors of some cancers. Nevertheless, its functional role and molecular mechanism remain obscure in LUAD. Our current study concentrates on exploring the biological function of FTX in LUAD.

**Methods:**

RT-qPCR was used to test the expression of FTX, miR-335-5p or NUCB2 in LUAD cells. The effect of FTX on LUAD progression was investigated by colony formation, EdU, flow cytometry, TUNEL, transwell and western blot assays. The interaction between microRNA-335-5p (miR-335-5p) and FTX or nucleobindin 2 (NUCB2) was confirmed by luciferase reporter assay.

**Results:**

RT-qPCR showed that FTX expression was up-regulated in LUAD cell lines. Loss-of-function assay indicated that FTX accelerated cell proliferation, migration and invasion, while inhibited cell apoptosis in LUAD. Besides, miR-335-5p, lowly expressed in LUAD cells, was discovered to be sponged by FTX. Subsequently, NUCB2 was identified as a target gene of miR-335-5p. Additionally, it was confirmed that NUCB2 functioned as an oncogene in LUAD. Rescue assays indicated that LUAD progression inhibited by FTX knockdown could be restored by NUCB2 up-regulation.

**Conclusion:**

FTX played an oncogenic role in LUAD and contributed to cancer development via targeting miR-335-5p/NUCB2 axis.

## Background

Lung cancer is one of the most aggressive tumors and its incidence and mortality demonstrated a swift growth in recent decades [1]. It can be histologically divided into small cell lung carcinoma (SCLC) and non-small cell lung carcinoma (NSCLC). As a predominant type of NSCLC, lung adenocarcinoma (LUAD) accounts for a quite important part of total lung cancer cases [2]. Although medical level has been improved, the clinical therapy of LUAD is still unsatisfactory because of late diagnosis, metastasis and recurrence [3]. Therefore, it is necessary to explore the potential biomarkers and molecular mechanism underlying LUAD for the improvement of the prognosis of LUAD patients.

Long noncoding RNAs (lncRNAs) are a group of RNAs longer than 200 nucleotides and have a limit in encoding proteins [4]. Substantial reports uncovered that abnormally-expressed lncRNAs are implicated in cancer occurrence and progression [5, 6]. For example, lncRNA DANCR is up-regulated in nasopharyngeal carcinoma and promotes metastasis through the interaction of NF90/NF45 complex [7]. LncRNA SNHG8 presents a high level, accelerates tumorigenesis and predicts tumor recurrence in hepatocellular carcinoma [8]. Previous studies showed that lncRNA could function as a competing endogenous RNA (ceRNA) and affect cancer progression by sponging microRNAs (miRNAs) to release messenger RNAs (mRNAs) at post-transcriptional level [9, 10]. For instance, lncRNA C5orf66-AS1 acts as a ceRNA in cervical cancer and promotes cancer cell proliferation by targeting miR-637/RING1 axis [11]. LncRNA CDKN2BAS serves as a ceRNA in hepatocellular carcinoma and predicts poor prognosis via regulating miR-153-5p/ARHGAP18 axis [12].

FTX, locating at X-chromosome inactivation (XCI) center, is a highly conserved lncRNA and exerts important role in the biological process of cancers [13]. FTX possesses an X-inactive-specific transcript and acts as the chief regulator of X-inactivation initiation. In addition, FTX was reported as a ceRNA and played oncogenic role in colorectal cancer [14] and gliomas [13]. Nevertheless, its function and regulatory mechanism have not been revealed in LUAD.

In this research, we probed the role and mechanism of lncRNA FTX in LUAD, and found that FTX was overexpressed in LUAD cells and contributed to LUAD progression by sequestering miR-335-5p and up-regulating NUCB2. This discovery provided a helpful theoretic basis for the exploration of LUAD therapeutic strategies.

## Materials and methods

### Cell lines

Human LUAD cell line (H1650, H1975, A549 and H1299) and human normal lung epithelial cell line (BEAS-2B) used in this study were propagated in the DMEM culture medium under 5% CO_2_ at 37 °C. The 10% FBS (Gibco, Waltham, MA) and 1% Pen/Strep solution were served as the supplements for DMEM. All cell lines were procured from the ATCC (Manassas, VA).

### RNA extraction and RT-qPCR

Total RNAs from H1650 and H1975 cells were obtained with the help of TRIZOL reagent (Invitrogen, Carlsbad, CA), and then converted into cDNA employing the PrimeScript RT reagent kit (Takara Bio, Tokyo, Japan). RT-qPCR procedure was achieved using SYBR Green PCR Master Mix (Takara), followed by 2^−ΔΔCT^ method analysis. Gene expression was relative to GAPDH or U6. The primers were shown in Table 1.

**Table 1:** The sequences for the PCR primers of genes

PCR primers		
Gene name	Forward primers	Reverse primers
FTX	GCCCATAGTGCTTCACTGGT	CATCCTTGCCTCAGAGGGA
miR-335-5p	TCAAGAGCAATAACGAAAAATGTG	CTCTACAGCTATATTGCCAGCCAC
NUCB2	GAACGTGTTACGAGTCAGTTTTTAGT	GACAACTGAGATCCAGAGAGGTAAG
U6	CTCGCTTCGGCAGCACA	AACGCTTCACGAATTTGCGT
GAPDH	TCCCATCACCATCTTCCA	CATCACGCCACAGTTTTCC

shRNA sequence	
sh-NC	CCGGCAGACATGATATCACATAGCTCTCGAGAGCTATGTGATATCATGTCTGTTTTTG
sh-FTX#1	CCGGCACTACATCTGGCTTACACTACTCGAGTAGTGTAAGCCAGATGTAGTGTTTTTG
sh-FTX#2	CCGGGACGTATACAGAATACTTCGTCTCGAGACGAAGTATTCTGTATACGTCTTTTTG
sh-NC	CCGGGAGTCAGTGTATAGTGCATAGCTCGAGCTATGCACTATACACTGACTCTTTTTG
sh-NUCB2	CCGGAACGTGTTACGAGTCAGTTTTCTCGAGAAAACTGACTCGTAACACGTTTTTTTG

### Transfection

The shRNAs specifically against FTX and NUCB2, as well as the pcDNA3.1/FTX and pcDNA3.1/NUCB2, were all produced by Genepharma (Shanghai, China). The negative control (NC) nonspecific shRNAs and empty pcDNA3.1 vectors were also acquired. In addition, miR-335-5p mimics/inhibitor and NC mimics/inhibitor were synthesized by Genechem (Shanghai, China). Cell transfection was implemented by application of the Lipofectamine 2000 (Invitrogen) for 48 h. The sequences were illustrated in Table 1.

### Colony formation

Clonogenic LUAD cells were cultivated for 14 days in the 6-well plates at 500 cells per well, and then treated with the 0.5% crystal violet solution for staining in 4% paraformaldehyde. Finally, colonies were counted manually.

### EdU incorporation assay

For EdU analysis, LUAD cells were cultivated for 3 h with the 100 ml of EdU incorporation assay kit (Ribobio, Guangzhou, China), and then treated with 4% paraformaldehyde for fixation. After permeabilization, DAPI solution was used to counterstain cell nuclei. Proliferative cells were determined under fluorescent microscope (Leica, Wetzlar, Germany).

### Cell cycle analysis

Cells with 48 h of transfection were collected, and then eluted with cold PBS. Fixed with 70% ethanol, transfected cells were treated with RNase A (100 μg/ml), followed by a staining of the propidium iodide solution. Incubated for 20 min, flow cytometry (BD Biosciences, San Jose, CA) was used to determine cell cycle distribution.

### Flow cytometer for apoptosis

Transfected LUAD cells were harvested for 15 min of double-staining with the Annexin V FITC/PI detection kit (Invitrogen) in the dark at 4 °C. The apoptotic rate of LUAD cells was determined by flow cytometer (BD Biosciences).

### TUNEL assay

Cell apoptosis was also detected by TUNEL assay in line with the protocol of One-Step TUNEL Apoptosis Assay Kit (Beyotime, Shanghai, China). After fixation and permeabilization, cells were treated with the DAPI solution, apoptotic cells were observed under fluorescent microscope.

### Transwell assays

The Matrigel-coated transwell chamber was procured from Corning Co (Corning, NY) for cell invasion analysis, while cell migration was analyzed without Matrigel coating. 5 × 10^3^ cells were placed into the upper chamber, while complete culture medium was added into the lower chamber. Invasive or migratory cells were fixed and stained with crystal violet 24 h later, and then counted under the microscope.

### In vivo assay and pulmonary metastasis assay

Male BALB/c nude mice (4-week-old) purchased from the Second Hospital of Tianjin Medical University were used for animal experiments with the ethical approval of the the Second Hospital of Tianjin Medical University. The BALB/C nude mice (five mice in a group) housed in a standard environment were subcutaneously inoculated with sh-FTX or sh-NC transfected H1650 cells (1 × 10^6^/mice). Then, tumor growth was monitored every 4 days and the mice were sacrificed by cervical dislocation after 28 days. Later, the xenograft tumors were removed and weighed. Finally, RT-qPCR was employed for the detection of FTX expression and western blot was performed to measure NUCB2 protein level. For pulmonary metastasis assay, stably transfected H1650 cells were injected into the tail vein of mice. Two weeks later, mice were sacrificed and the lungs were dissected. Then, the metastatic nodules were counted.

### HE staining

For HE staining, sections were deparaffinized and rehydrated, and then incubated with hematoxylin. After staining in acid ethanol and eosin with five dips, the sections were dehydrated and cleared. Finally, the images were obtained with a fluorescence microscope.

### Western blot

The cellular total proteins were extracted from LUAD cells for treating with the 12% SDS-PAGE gel and shifting onto the PVDF membranes. After adding 5% skimmed milk, the primary antibodies against the internal control GAPDH and slug, Vimentin, N-cadherin, E-cadherin, NUCB2, p-AKT, AKT, p-mTOR, mTOR, p-STAT3, STAT3 as well as the appropriate HRP-tagged secondary antibodies (all, Abcam, Cambridge, MA) were diluted and used for probing membranes. The blots were monitored by enhanced chemiluminescence reagent (Santa Cruz Biotechnology, Santa Cruz, CA).

### FISH

The fixed LUAD cells were collected and air-dried for incubation in the hybridization buffer with the FTX FISH probe (Ribobio). Following adding the Hoechst staining solution, stained samples were assayed under fluorescent microscope.

### RNA pull down

The cellular protein samples from LUAD cells were acquired in RIPA lysis buffer and incubated with the wild-type (WT) or mutated (Mut) biotinylated FTX probes for 1 h at 4 °C. The streptavidin agarose magnetic beads were added to collect the pull-downs. The miRNA enrichments were analyzed by RT-qPCR.

### Dual-luciferase reporter assays

Fragments of FTX or NUCB2 sequences which contained the miR-335-5p binding sites were amplified by RT-PCR and then cloned into pmirGLO reporter vectors (Promega, Madison, WI) to generate FTX-WT/NUCB2-WT luciferase reporters. The mutations were constructed by Site-Directed Mutagenesis kit (Invitrogen) and named as FTX-Mut and NUCB2-/Mut. The recombinant reporter vectors were co-transfected into LUAD cells with indicated transfection plasmids for 48 h, then Dual-Luciferase Reporter Assay System (Promega) was applied for luciferase activities.

### RNA immunoprecipitation (RIP)

1 × 10^7^ LUAD cells were reaped from the RIP lysis buffer for cultivating with the beads-bound anti-Ago2 or anti-IgG antibody (Millipore, Bedford, MA) in the RIP buffer. The RNA levels in the precipitates were estimated using RT-qPCR.

### Statistical analyses

Results were presented as the mean ± standard deviation (SD) of more than three independent bio-repeats. Data analysis in each group was processed by student’s t-test or one-way analysis of variance (ANOVA) by application of PRISM 6 software (GraphPad, San Diego, CA). The p-value less than 0.05 was set as the threshold of significant level.

## Results

### FTX was overexpressed and enhanced the progression in LUAD

**Fig. 1:**
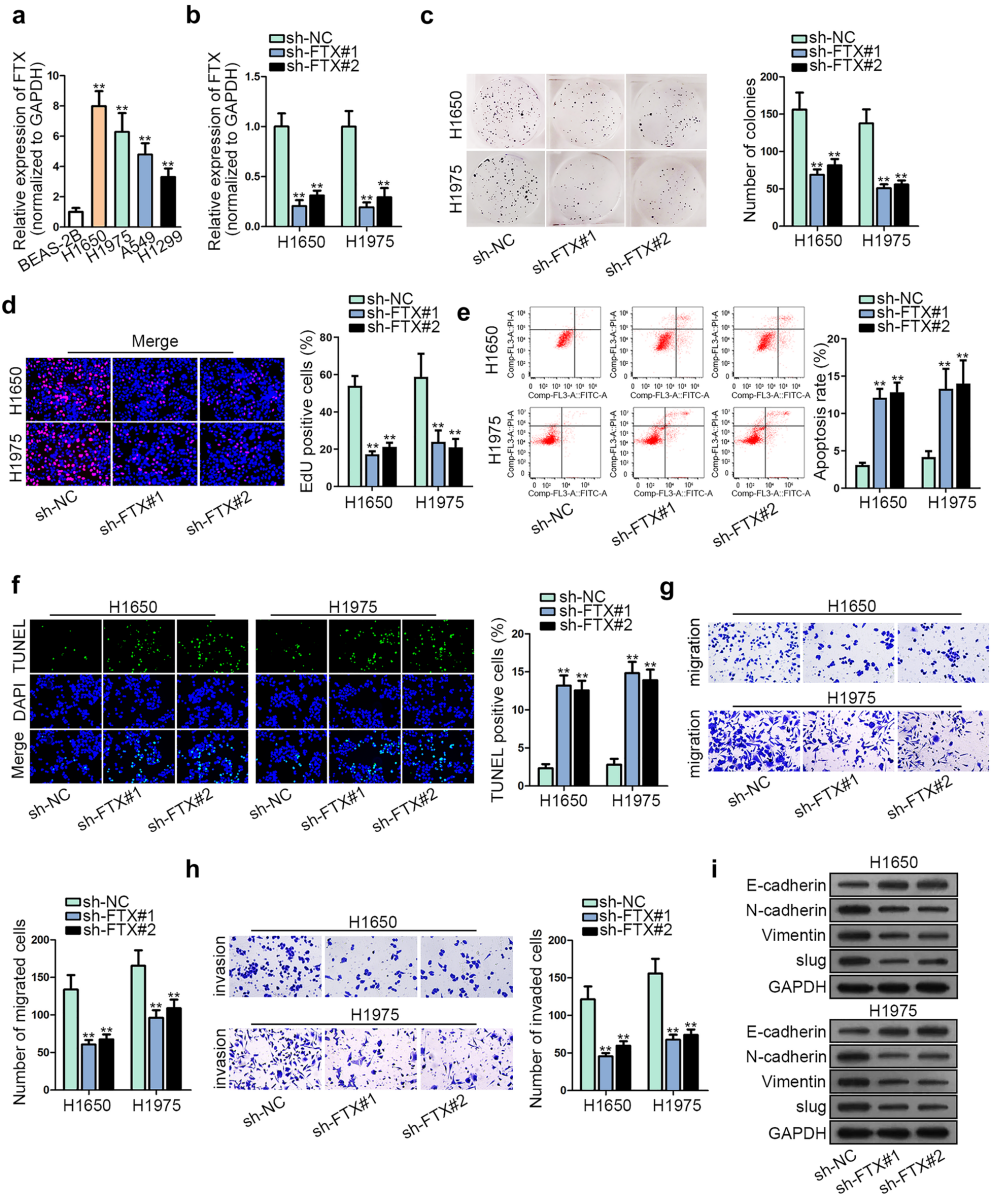
FTX was overexpressed and enhanced the progression in LUAD. **a** FTX expression in LUAD cell lines and BEAS-2B cell line. **b** FTX knockdown efficacy in H1650 and H1975 was determined by RT-qPCR. **c**, **d** Proliferation of FTX-depleted LUAD cells was assessed by colony formation and EdU assays. **e**, **f** Flow cytometry analysis and TUNEL assays were applied for evaluating cell apoptosis under FTX knockdown. **g**, **h** LUAD cell migration and invasion were analyzed after FTX was silenced through transwell assay. **i** Western blot was used to appraise EMT-related proteins levels in cells transfected with sh-FTX or sh-NC. **p < 0.01

To explore the expression pattern of FTX in LUAD, we detected FTX expression in four LUAD cell lines (H1650, H1975, A549 and H1299). The human normal lung epithelial cell line (BEAS-2B) was seen as a control. As a result, FTX was highly expressed in LUAD cell lines, particularly in H1650 and H1975 cells (Fig. 1a), suggesting the aberrant expression of FTX in LUAD. Therefore, we explored the biological function of FTX in LUAD by designing loss-of-function assay. Before the experiment, FTX expression was stably silenced in H1650 and H1975 cells (Fig. 1b). It was well known that cell proliferation and apoptosis are the important processes involved in cancer progression. Therefore, the effect of FTX on LUAD cell proliferation and apoptosis was assessed. Colony formation assay and EdU assay manifested that FTX silencing notably lessened the LUAD proliferation of H1650 and H1975 cells (Fig. 1c, d). Further, cell cycle analysis showed that silenced FTX remarkably ascended the proportion of H1650 and H1975 cells in G0/G1 phases, and reduced cell proportion in S and G2/M phases (Additional file 1: Figure S1A). Later, we viewed that cell apoptosis was potently boosted in sh-FTX transfected H1650 and H1975 cells (Fig. 1e, f and Additional file 1: Figure S1B). Previous studies indicated that cancer metastasis was dependent on the abilities of cell migration and invasion [7, 15]. Based on this, we investigated the biological role of FTX in LUAD by testing migratory and invasive capacities of H1650 and H1975 cells. Results of transwell assay unveiled that knockdown of FTX conspicuously hindered cell migration and invasion in LUAD (Fig. 1g, h). Since epithelial-mesenchymal transition (EMT) process is closely correlated with cell migration and invasion [16, 17], we evaluated whether FTX could induce EMT in LUAD cells. Western blot assay implied that protein level of epithelial marker (E-cadherin) was obviously ascended while protein levels of mesenchymal markers (N-cadherin and Vimentin) and EMT transcription factor (slug) were evidently alleviated in H1650 and H1975 cells transfected with sh-FTX (Fig. 1i). To be concluded, FTX acted as an oncogene in LUAD.

### FTX sponged miR-335-5p in LUAD

**Fig. 2:**
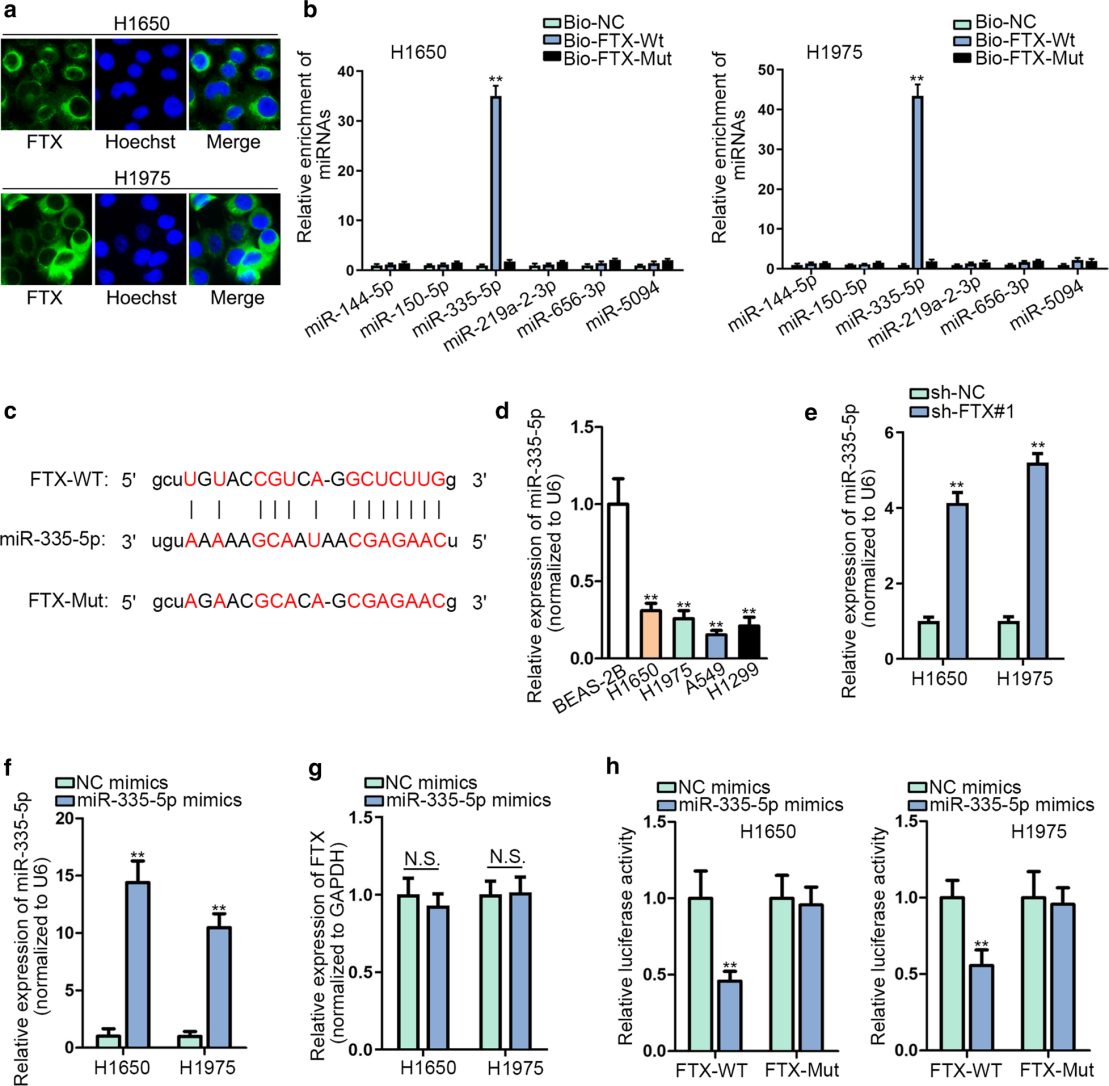
FTX sponged miR-335-5p in LUAD. **a** The location of FTX in LUAD cells was showed by FISH assay. **b** RNA pull-down assay examined the interaction between FTX and predicted miRNAs. **c** Binding sites between FTX and miR-335-5p. **d** MiR-335-5p expression was detected by RT-qPCR in LUAD cell lines and BEAS-2B cell line. **e** MiR-335-5p expression was detected in sh-FTX transfected LUAD cells. **f** MiR-335-5p up-regulation efficiency was examined in H1650 and H1975 cells. **g** Effect of miR-335-5p mimics on FTX expression was tested. **h** Luciferase activity of FTX-WT/Mut reporters was measured in LUAD cells transfected with miR-335-5p mimics or NC mimics. **p < 0.01, N.S. showed no significance

Subsequently, we deciphered molecular mechanism that FTX involved in LUAD. It has been reported that lncRNA could regulate cancer progression by serving as miRNA sponge in the cytoplasm [9, 10]. Thus, we detected the localization of FTX in LUAD cells by FISH assay, and results displayed that FTX was abundantly distributed in LUAD cell cytoplasm (Fig. 2a), giving the potential of ceRNA hypothesis. Using starBase (http://starbase.sysu.edu.cn), 6 miRNAs were predicted to possess complementary bases on the sequence of FTX. To further filtrate the selection, RNA pull down assay was performed. Results revealed that biotinylated wild-type FTX pulled down miR-335-5p while other miRNAs were not affected (Fig. 2b). Besides, the binding site between FTX and miR-335-5p was projected and the mutation was constructed (Fig. 2c). Moreover, we found that miR-335-5p expressed at a low level in LUAD cells (Fig. 2d). To explore the relationship between miR-335-5p and FTX, we tested miR-335-5p expression in sh-FTX transfected cells. Results disclosed that miR-335-5p expression was elevated upon FTX depletion (Fig. 2e). Then, the transfection efficiency of miR-335-5p mimics was examined. Results of RT-qPCR displayed that miR-335-5p expression achieved an escalation by transfecting miR-335-5p mimics in LUAD cells (Fig. 2f). Interestingly, we found that the expression of FTX was not affected by up-regulated miR-335-5p (Fig. 2g). To further confirm the interaction between FTX and miR-335-5p, luciferase reporter assay was exploited. As we observed, the transfection of miR-335-5p mimics led to a decreased luciferase activity of FTX-WT vector while no evident changes were seen in FTX-Mut vector (Fig. 2h). Collectively, FTX acted as a sponge of miR-335-5p in LUAD.

### MiR-335-5p down-regulation restored the influence of FTX knockdown on LUAD cells

**Fig. 3:**
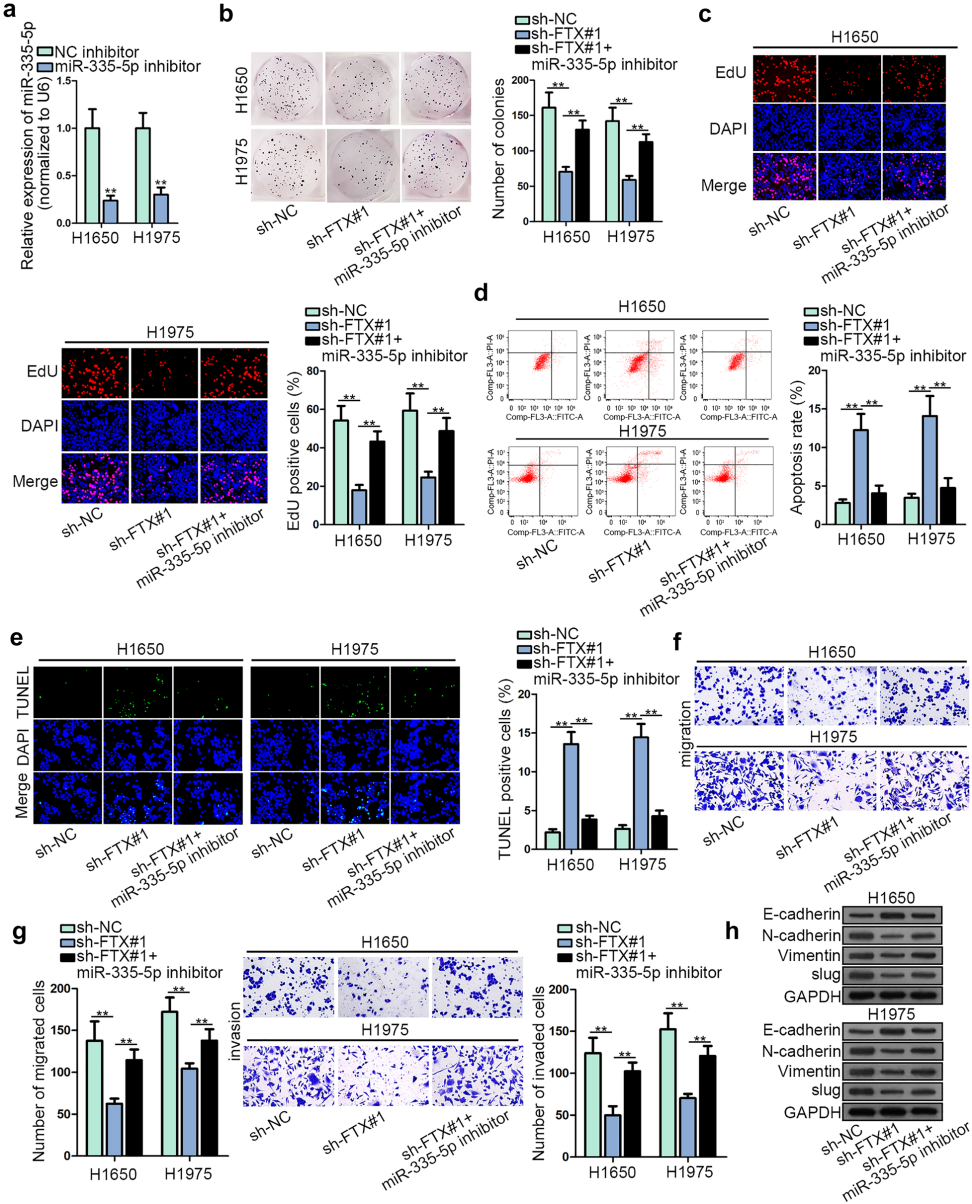
MiR-335-5p down-regulation restored the influence of FTX knockdown on LUAD cells. **a** Transfection efficiency of miR-335-5p inhibitor was determined in H1650 and H1975 cells. **b**, **c** The proliferation in LUAD cells transfected with sh-NC, sh-FTX, sh-FTX + miR-335-5p inhibitor was confirmed via colony formation assay and EdU assay. **d**, **e** Flow cytometry analysis and TUNEL assay were conducted to analyze cell apoptosis in the groups of sh-FTX sh-FTX + miR-335-5p inhibitor or sh-NC. **f**, **g** Transwell assay appraised migratory and invasive abilities in cells transfected with indicated plasmids. **h** Western blot assay was used to appraise the level of EMT-relevant proteins in each group. **p < 0.01

Here, we checked whether FTX boosted LUAD progression by sponging miR-335-5p. Hence, some rescue assays were designed and conducted. Firstly, the plasmid of miR-335-5p inhibitor was transfected into LUAD cells to repress miR-335-5p expression (Fig. 3a). In colony formation and EdU assays, miR-335-5p depletion could abrogate FTX knockdown-mediated suppression on LUAD cell proliferative capacity (Fig. 3b, c and Additional file 1: Figure S1C). In addition, the recovering effect of down-regulated miR-335-5p on the apoptosis increased by FTX down-regulation was seen in flow cytometry analysis and TUNEL assay (Fig. 3d, e and Additional file 1: Figure S1D). According to transwell assays, declined migratory and invasive abilities resulting from silenced FTX were abrogated by miR-335-5p repression (Fig. 3f, g). Data of western blot assay delineated that the effect of FTX deficiency on the protein levels of E-cadherin, N-cadherin, Vimentin and slug was countervailed by miR-335-5p suppression (Fig. 3h). To sum up, miR-335-5p down-regulation restored the influence of FTX knockdown on LUAD cell progression.

### NUCB2 was a target of miR-335-5p and acted as an oncogene in LUAD

**Fig. 4:**
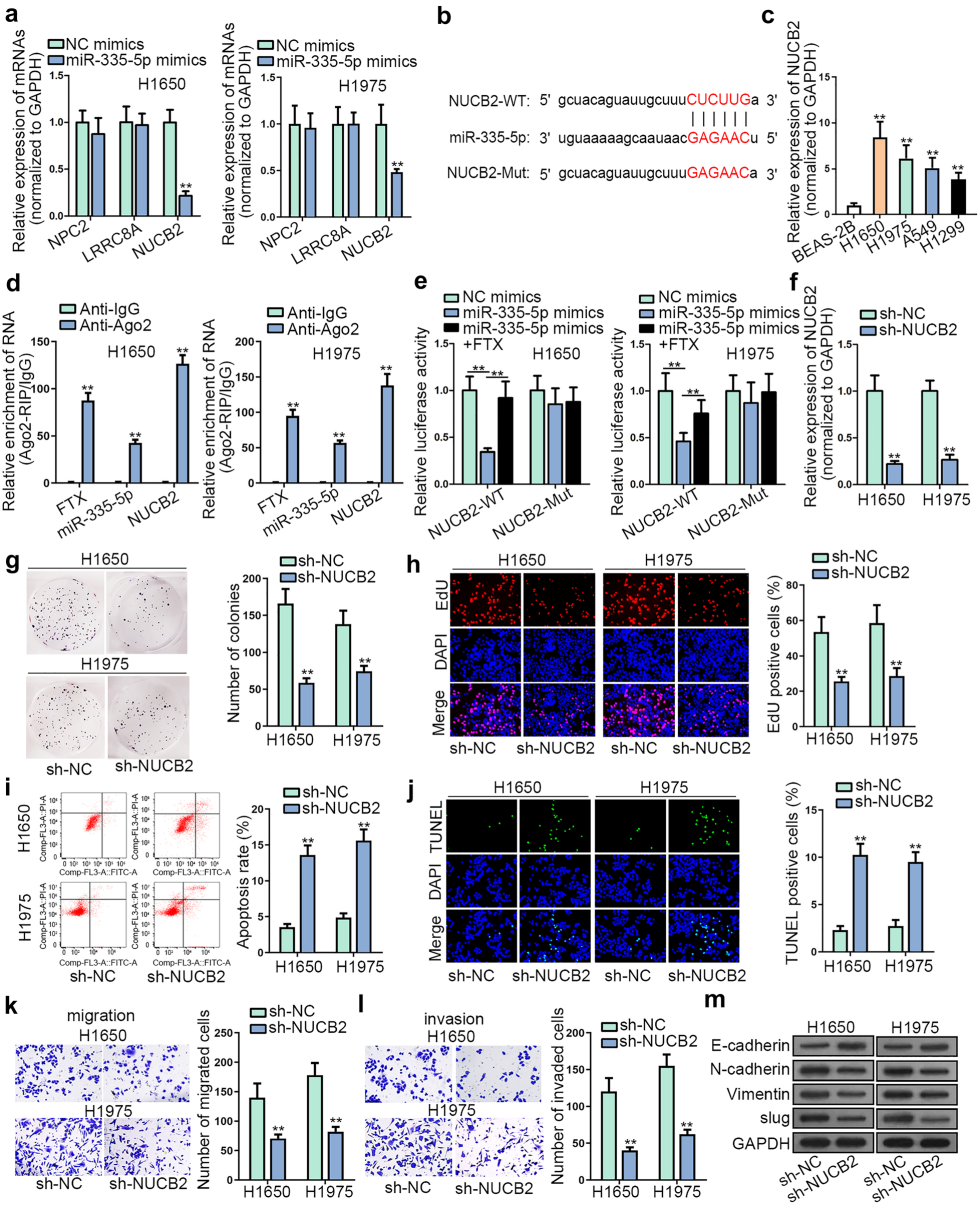
NUCB2 was a target of miR-335-5p and acted as an oncogene in LUAD. **a** Effect of miR-335-5p mimics on the expressions of predicted mRNAs was validated through RT-qPCR. **b** MiR-335-5p was predicted to possess a binding site on NUCB2. **c** NUCB2 expression was assessed in LUAD cell lines and BEAS-2B cell line was seen as a control. **d** RIP assay testified the enrichment of FTX, miR-335-5p and NUCB2 in RISC. **e** Luciferase activity of NUCB2-WT/Mut in response to the indicated plasmids was certificated via luciferase reporter assay. **f** NUCB2 knockdown efficacy in H1650 and H1975 cells. **g**, **h** The influence of sh-NUCB2 or sh-NC on LUAD cell proliferation was assessed by colony formation and EdU assays. **i**, **j** The apoptosis in sh-NUCB2 or sh-NC group was evaluated via flow cytometry analysis and TUNEL assay. **k**, **l** Transwell assay was performed to appraise cell migration and invasion in sh-NUCB2 or sh-NC group. **m** Western blot assay was conducted to evaluate EMT-relevant protein levels in sh-NUCB2 or sh-NC group. **p < 0.01

With the purpose of supporting ceRNA hypothesis, the targets of miR-335-5p were explored. Through starBase (an online tool), NPC2, LRRC8A and NUCB2 were predicted with the strict condition (pan-cancer ≥ 8; degradome data ≥ 3 and clip data ≥ 5). To select the optimal gene for miR-335-5p in LUAD, the expressions of these mRNAs were measured in LUAD cells transfected with miR-335-5p mimics or NC mimics. As a result, NUCB2 expression was inhibited by up-regulated miR-335-5p while no apparent changes were observed in the other mRNAs (Fig. 4a). Besides, the complementary binding site between miR-335-5p and NUCB2 was predicted (Fig. 4b). Furthermore, NUCB2 was found at a high level in LUAD cell lines (Fig. 4c). It has been reported that Ago2 is the core component of RNA-induced silencing complex (RISC) and miRNAs could exert the effect of gene silencing by RISC [18]. To assess whether FTX, miR-335-5p and NUCB2 co-exist in the identical RISC, RIP assay was carried out. Results disclosed that FTX, miR-335-5p and NUCB2 were all abundant in the beads conjugated with Ago2 antibody but not IgG antibody (Fig. 4d). Later, it was exhibited in luciferase reporter assay that miR-335-5p mimics abated the luciferase activity of NUCB2-WT reporter rather than NUCB2-Mut reporter. In addition, the decreased luciferase activity was recovered by pcDNA3.1/FTX (Fig. 4e). Above results uncovered that NUCB2 was a target of miR-335-5p in FTX-mediated ceRNA network. Subsequently, the functional role of NUCB2 in LUAD was assessed. The expression of NUCB2 was reduced in LUAD cells by transfecting sh-NUCB2 (Fig. 4f). We found that NUCB2 depletion weakened the proliferative ability of LUAD cells (Fig. 4g, h). Meanwhile, LUAD cell apoptosis was stimulated by sh-NUCB2 transfection (Fig. 4i, j and Additional file 1: Figure S1E). Moreover, cell migration, invasion and EMT were refrained upon NUCB2 knockdown (Fig. 4k–m). In addition, as we all known, many signals could control the above processes; thus, we explored the downstream signals of NUCB2. NUCB2 was reported to be associated with AKT/mTOR pathway and STAT3 pathway [19, 20]. As shown in Additional file 1: Figure S1F, FTX knockdown significantly decreased the levels of phosphorylated AKT and mTOR, and NUCB2 rescued the effect of silenced FTX. However, no detectable changes were observed in phosphorylated STAT3 and total levels of AKT, mTOR and STAT3, which suggested that NUCB2 could mediate AKT/mTOR rather than STAT3 pathway in LUAD. In summary, NUCB2 was a target of miR-335-5p and acted as an oncogene in LUAD.

### FTX promoted LUAD progression through increasing NUCB2 expression

**Fig. 5:**
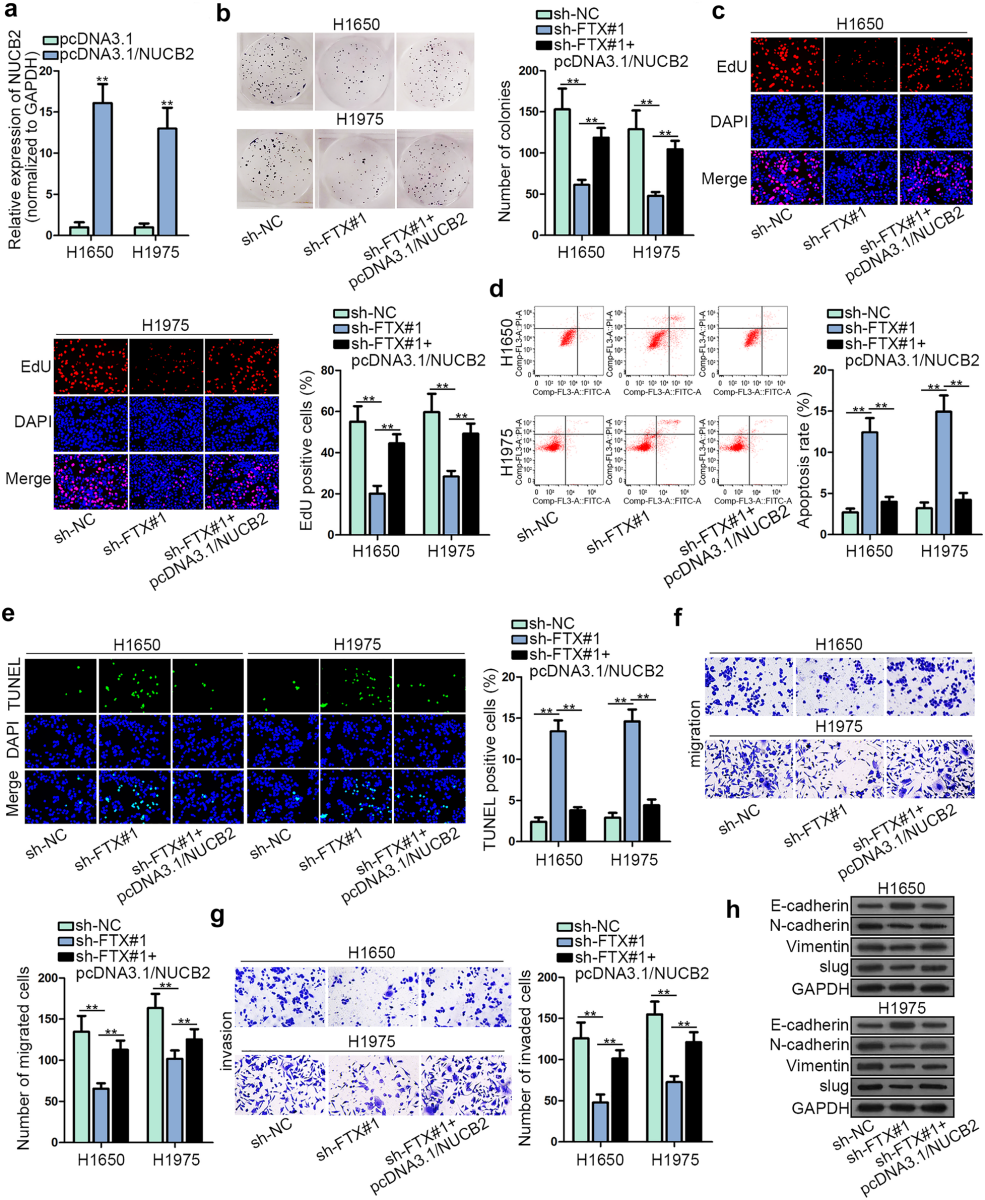
FTX promoted LUAD progression through increasing NUCB2 expression. **a** Transfection efficiency of pcDNA3.1/NUCB2 was validated using RT-qPCR. **b**, **c** LUAD cell proliferation was verified in each group. **d**, **e** The apoptosis of cells transfected with appointed plasmids was tested. **f**, **g** The migration and invasion in cells transfected with the appointed plasmids were assessed. **h** EMT-related protein levels were detected with the transfection of indicated plasmids. **p < 0.01

In order to further check the function of FTX/miR-335-5p/NUCB2 axis in LUAD, rescue assays were designed and performed. At the beginning, we elevated NUCB2 expression by transfecting pcDNA3.1/NUCB2 in LUAD cells (Fig. 5a). Colony formation and EdU assays certified that down-regulation of FTX exceedingly curbed cell proliferation in LUAD while pcDNA3.1/NUCB2 counteracted FTX silencing-imposed effect (Fig. 5b, c). Additionally, NUCB2 overexpression was also released G0/G1 arrest of LUAD cells caused by FTX depletion (Additional file 1: Figure S1G). In terms of LUAD cell apoptosis, FTX depletion-hastened apoptosis was neutralized by pcDNA3.1/NUCB2, as shown in flow cytometry analysis and TUNEL assay (Fig. 5d, e and Additional file 1: Figure S1H). The suppressed migratory and invasive abilities caused by down-regulation of FTX were recovered by overexpression of NUCB2 (Fig. 5f, g). Western blot assay pictured that escalation of E-cadherin protein level and decrease of N-cadherin, slug, and Vimentin protein levels caused by FTX knockdown could be counterbalanced by pcDNA3.1/NUCB2 (Fig. 5h). To summarize, FTX accelerated LUAD progression via lifting NUCB2 expression in vitro. Then, we investigated whether FTX played the same role in vivo. Nude mice were subcutaneously inoculated with H1650 cells stably transfected with sh-FTX or sh-NC. Images of tumors in sh-FTX and sh-NC groups were exhibited (Additional file 2: Figure S2A). Tumor volume and weight were respectively detected and evaluated. FTX knockdown reduced the volume and weight of tumors compared with sh-NC group (Additional file 2: Figure S2B, C). Then we assessed the expression of FTX and found that FTX expression was reduced due to FTX deficiency (Additional file 2: Figure S2D). Owing to the accelerative effect of FTX on cell migration and invasion, we determined the role of FTX deficiency in cell metastasis in vivo. Consistently, lung metastasis of mice was significantly hindered in sh-FTX group compared with sh-NC group (Additional file 2: Figure S2E). Finally, western blot assay examined that NUCB2 protein level was restrained under FTX silencing (Additional file 2: Figure S2F). Therefore, we concluded that FTX promoted LUAD progression through increasing NUCB expression.

## Discussion

LUAD is an aggressive tumor with a high occurrence rate and mortality, and various dysregulated lncRNAs were reported to be associated with LUAD progression. For instance, lncRNA LIN28B-AS1 promotes the proliferation of LUAD cells [21]. LncRNA NEAT1 stimulates cell migration and hampers cell apoptosis in LUAD [22]. LncRNA SOX21-AS1 exacerbates cell proliferation and indicates a poor prognosis in LUAD [23]. LncRNA FTX was observed to be up-regulated in glioma and boost cell proliferation and invasion [13]. Nevertheless, it has not been studied in LUAD. Thus, we were the first to discover the high level of FTX in LUAD cell lines. Tumor growth and metastasis involve multiple cellular activities, such as proliferation, cell cycle, apoptosis, migration, invasion, and EMT [24, 25]. Herein, loss-of-function assay disclosed that silenced FTX inhibited cell proliferation by inducing cell cycle arrest and cell apoptosis, indicating the role of FTX in LUAD cell growth. Accordingly, we affirmed that FTX silence hindered LUAD tumorigenesis in vivo. Furthermore, FTX knockdown also repressed the metastasis-associated biological events in LUAD in vitro, including cell migration, invasion and EMT process in LUAD, and in vivo analysis confirmed that FTX1 deficiency decreased metastatic nodules. All results pointed out the oncogenic property of FTX in LUAD.

Recently, existing evidence has proposed a new regulatory mechanism that lncRNAs act as ceRNAs in the cytoplasm through competitively binding miRNAs to seclude their suppressive effect on target mRNAs [10]. Specifically, numerous miRNAs, participating in LUAD development, were reported to be regulated by lncRNAs [26–28]. FTX has been supported as a ceRNA in several tumors, such as hepatocellular carcinoma [29], osteosarcoma [30] and colorectal cancer [14]. Herein, our present study first showed that FTX was a cytoplasmic RNA and interacted with miR-335-5p in LUAD. Prior studies demonstrated that miR-335-5p was a tumor suppressor and involved in ceRNA network in renal cell carcinoma [31], gastric cancer [32] and osteosarcoma [33]. Here, miR-335-5p was lowly expressed in LUAD cell lines and could combine with FTX. In addition, the inhibitory role of down-regulated FTX in LUAD cell progression could be antagonized by miR-335-5p inhibition. In conclusion, our research expounded that FTX was a molecular sponge of miR-335-5p.

Nucleobindin 2 (NUCB2) was featured as active regulators in the biological movements [34, 35]. In colorectal cancer, up-regulation of NUCB2 was reported to associate with metastasis and aggravate the carcinoma progression [36]. Additionally, NUCB2 was up-regulated in prostate cancer and served as a prognostic biomarker [37]. Our data first reported that NUCB2 was a downstream target of miR-335-5p and high NUCB2 expression was detected in LUAD cell lines. Functional assay conformed that NUCB2 played the oncogenic role in LUAD. Rescue experiments suggested that NUCB2 overexpression could counter repressive effect of silenced FTX on LUAD progression. Additionally, previous studies showed that NUCB2 can regulate several pathways, including AKT/mTOR and STAT3 pathway [19, 20]. Herein, we found that NUCB2 axis could affect AKT/mTOR pathway by changing Ser473 (AKT phosphorylation site) and Ser2448 (mTOR phosphorylation site) but cannot affect STAT3 pathway. Although a study showed that UNCB2 affected both p-mTOR and p-STAT3 in hepatic gluconeogenesis [20], our data indicated that in NUCB2 cannot jointly regulate STAT3 and mTOR signaling in LUAD cells, probably because that in different cell types, the mechanisms and links between pathways and regulatory molecules vary. However, importantly, AKT/mTOR pathway is widely recognized as a pathway regulating cell growth and metastasis [38, 39], so it is reasonable to deduce that AKT/mTOR might be a downstream signaling for FTX/miR-335-5p/NUCB2 in LUAD. The detailed mechanism will be explored in the future for better explanation.

## Conclusion

Conclusively, lncRNA FTX was an oncogene in LUAD and acted as a ceRNA to drive LUAD progression by targeting miR-335-5p/NUCB2 axis and mediating AKT/mTOR pathway, providing a helpful theoretic basis to explore potential therapeutic strategies for the patients suffered from LUAD. Although we have explored AKT/mTOR pathway in our study, further exploration on the downstream signals will be conducted in the future.

## Supplementary information

**Additional file 1: Figure S1.** (A) Cell cycle was analyzed with the transfection of sh-FTX or sh-NC. (B) The apoptosis of LUAD cells in sh-FTX or sh-NC group. (C) The proliferation of H1650 cells in sh-FTX, sh-FTX + miR-335-5p inhibitor or sh-NC group. (D) The apoptosis of LUAD cells in sh-FTX, sh-FTX + miR-335-5p inhibitor or sh-NC group. (E) The apoptosis of LUAD cells in sh-NUCB2 or sh-NC group. (F) The protein levels of p-AKT, AKT, p-mTOR, mTOR, p-STAT3, STAT3 in sh-FTX, sh-FTX + pcDNA3.1/NUCB2 or sh-NC group was detected. (G) The analysis of cell cycle in sh-FTX, sh-FTX + pcDNA3.1/NUCB2 or sh-NC group. (H) The apoptosis of LUAD cells in sh-FTX, sh-FTX + pcDNA3.1/NUCB2 or sh-NC group. **P < 0.01.

**Additional file 2: Figure S2.** (A) Images of tumors removed from the mice transplanted with sh-FTX or sh-NC transfected H1650 cells. (B-C) Tumor volume and tumor weight in sh-FTX or sh-NC groups were quantified. (D) Expression of FTX under the transfection of sh-FTX was determined by RT-qPCR. (E) HE staining was used to confirm the metastatic lung nodules. (F) NUCB2 protein level was evaluated after silencing of FTX using western blot assay. **P < 0.01.

## Data Availability

Not applicable.

## Abbreviations

lncRNA: Long noncoding RNA
LUAD: Lung adenocarcinoma
FTX: FTX transcript, XIST regulator
miR-335-5p: microRNA-335-5p
NUCB2: Nucleobindin 2
SCLC: Small cell lung carcinoma
NSCLC: Non-small cell lung carcinoma
ceRNA: Competing endogenous RNA
miRNA: microRNA
mRNA: Messenger RNA
DMEM: Dulbecco’s Modified Eagle’s Medium
FBS: Fetal bovine serum
RT-qPCR: Reverse transcription quantitative polymerase chain reaction
GAPDH: Glyceraldehyde-3-phosphate dehydrogenase
shRNA: Short hairpin RNA
NC: Negative control
EdU: 5-Ethynyl-2′-deoxyuridine
SDS-PAGE: Sodium dodecyl sulfate-polyacrylamide gel electrophoresis
PVDF: Polyvinylidene fluoride
RIP: RNA immunoprecipitation
IgG: Immunoglobulin G
SD: Standard deviation
ANOVA: One-way analysis of variance
RISC: RNA-induced silencing complex
EMT: Epithelial–mesenchymal transition
FISH: Fluorescent in situ hybridization
