# Supplementary figures and images for: FTX contributes to cell proliferation and migration in lung adenocarcinoma via targeting miR-335-5p/NUCB2 axis
Source: Cancer Cell Int. 2020 Mar 23;20:89. doi: 10.1186/s12935-020-1130-5 (PMC7092578; doi:10.1186/s12935-020-1130-5)

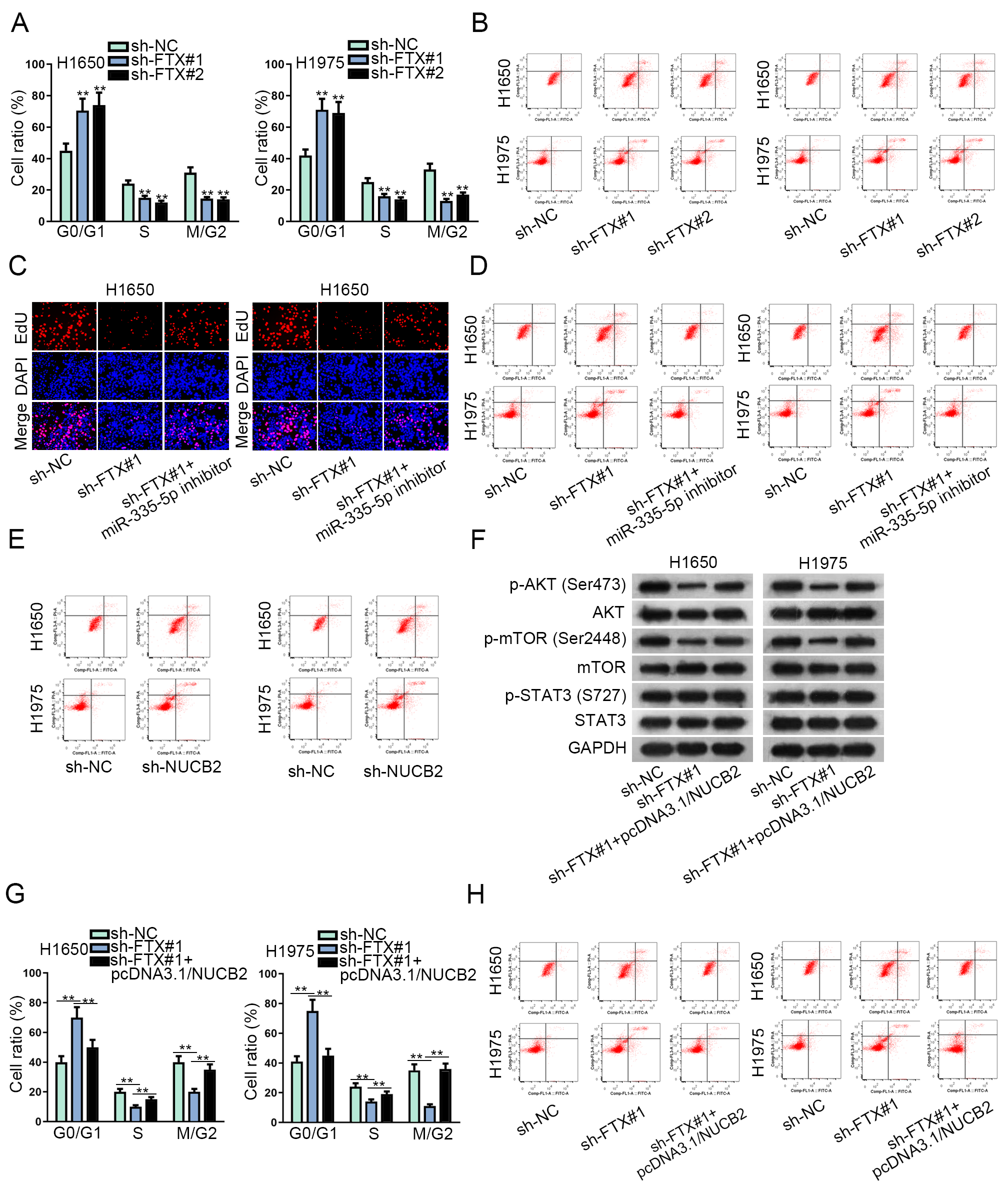

Supplement: Supplementary file 1 — Additional file 1: Figure S1. (A) Cell cycle was analyzed with the transfection of sh-FTX or sh-NC. (B) The apoptosis of LUAD cells in sh-FTX or sh-NC group. (C) The proliferation of H1650 cells in sh-FTX, sh-FTX + miR-335-5p inhibitor or sh-NC group. (D) The apoptosis of LUAD cells in sh-FTX, sh-FTX + miR-335-5p inhibitor or sh-NC group. (E) The apoptosis of LUAD cells in sh-NUCB2 or sh-NC group. (F) The protein levels of p-AKT, AKT, p-mTOR, mTOR, p-STAT3, STAT3 in sh-FTX, sh-FTX + pcDNA3.1/NUCB2 or sh-NC group was detected. (G) The analysis of cell cycle in sh-FTX, sh-FTX + pcDNA3.1/NUCB2 or sh-NC group. (H) The apoptosis of LUAD cells in sh-FTX, sh-FTX + pcDNA3.1/NUCB2 or sh-NC group. **P < 0.01. [file 12935_2020_1130_MOESM1_ESM.tif]

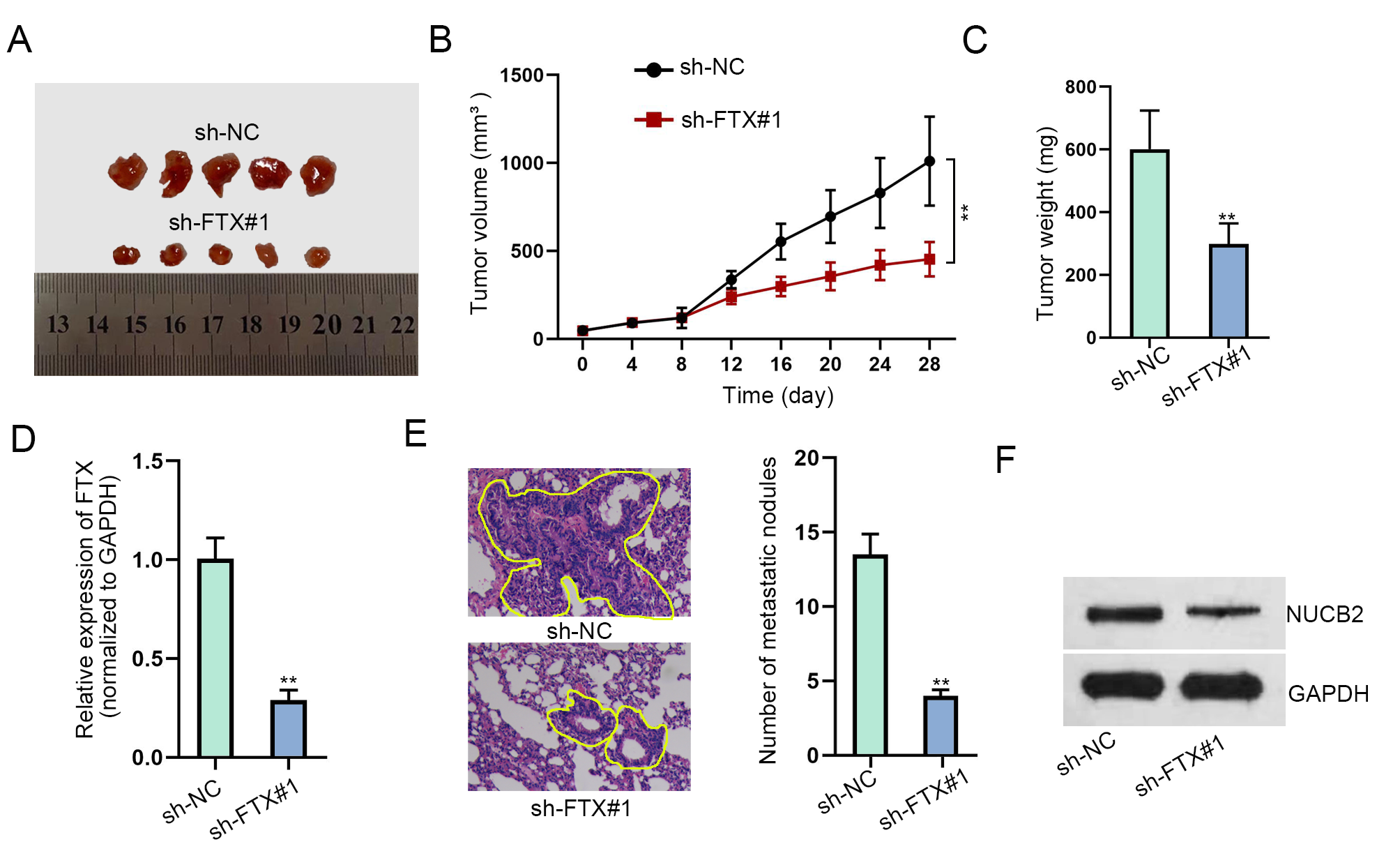

Supplement: Supplementary file 2 — Additional file 2: Figure S2. (A) Images of tumors removed from the mice transplanted with sh-FTX or sh-NC transfected H1650 cells. (B-C) Tumor volume and tumor weight in sh-FTX or sh-NC groups were quantified. (D) Expression of FTX under the transfection of sh-FTX was determined by RT-qPCR. (E) HE staining was used to confirm the metastatic lung nodules. (F) NUCB2 protein level was evaluated after silencing of FTX using western blot assay. **P < 0.01. [file 12935_2020_1130_MOESM2_ESM.tif]
